# Supplementary figures and images for: Tumor suppressor p73 induces apoptosis of murine peritoneal cell after exposure to hydatid cyst antigens; a possibly survival mechanism of cystic echinococcosis in vivo mice model
Source: PLoS One. 2023 Oct 5;18(10):e0292434. doi: 10.1371/journal.pone.0292434 (PMC10553360; doi:10.1371/journal.pone.0292434)

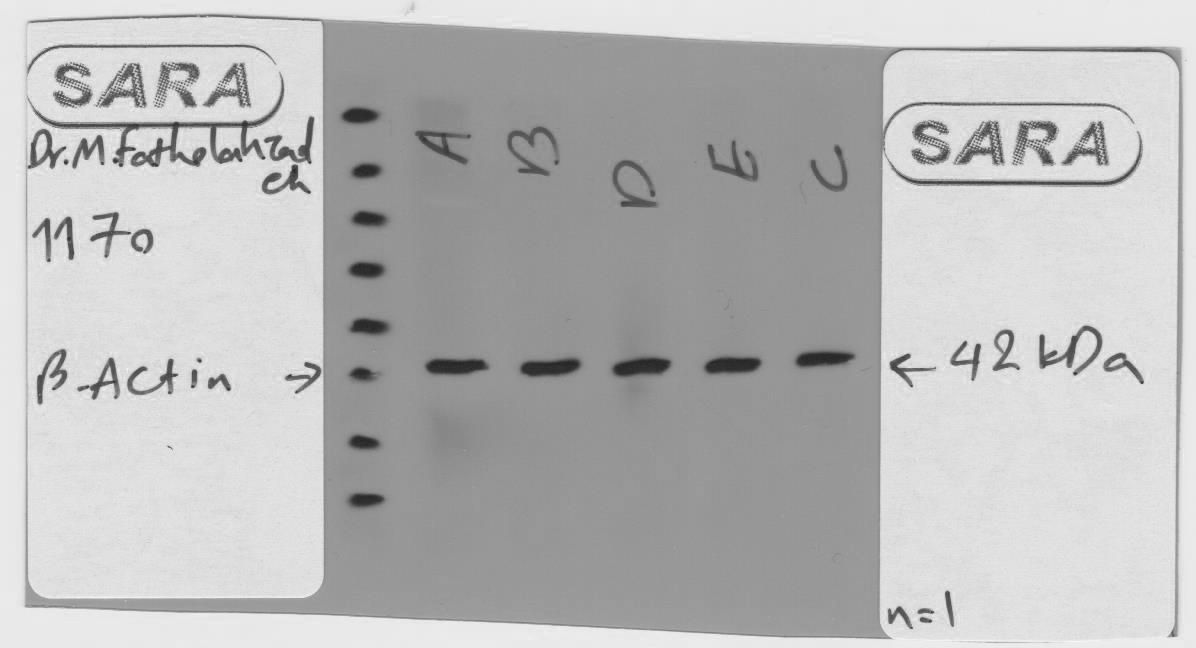

Supplement: S1 Fig — (JPG) [file pone.0292434.s001.jpg]

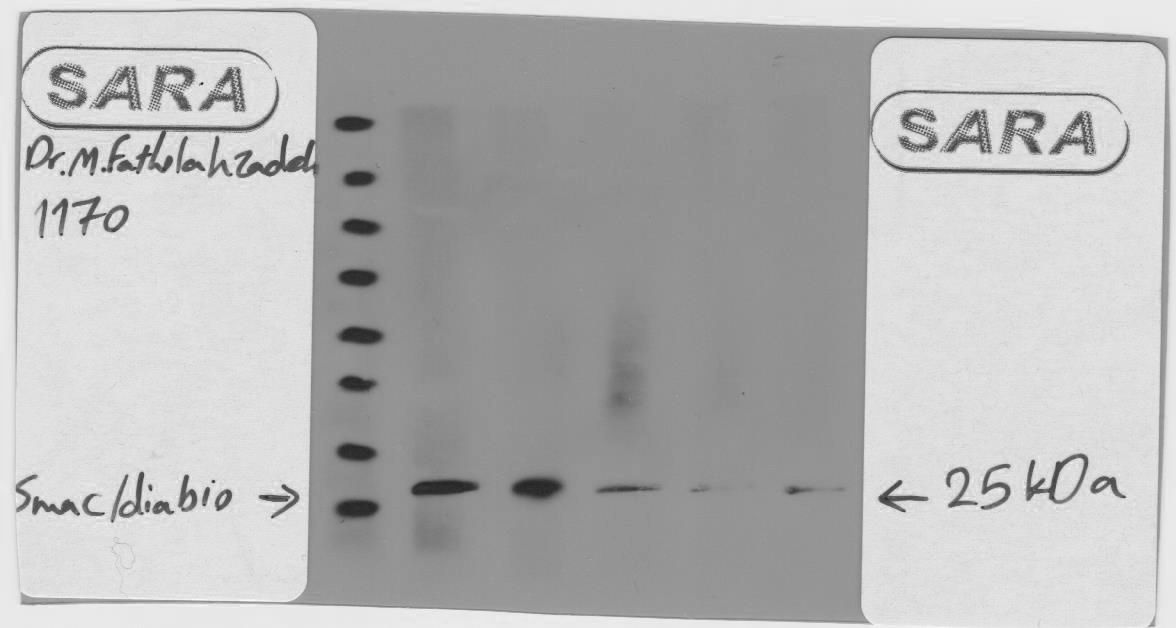

Supplement: S2 Fig — (JPG) [file pone.0292434.s002.jpg]

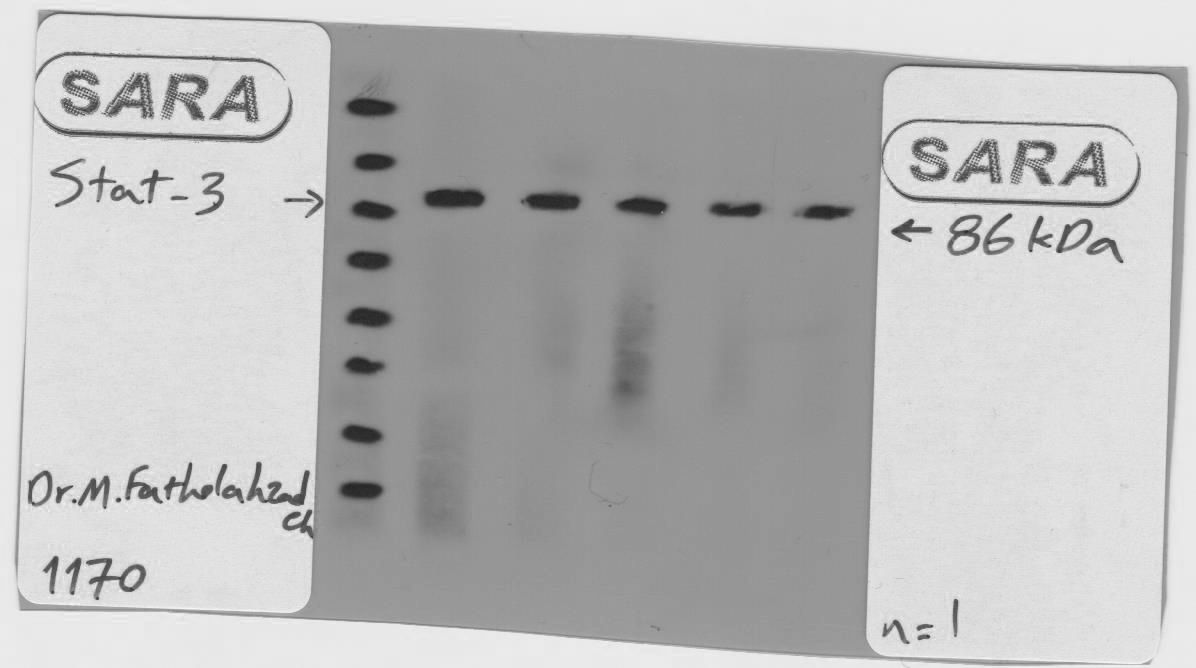

Supplement: S3 Fig — (JPG) [file pone.0292434.s003.jpg]
