# Supplementary material for: Tumor suppressor p73 induces apoptosis of murine peritoneal cell after exposure to hydatid cyst antigens; a possibly survival mechanism of cystic echinococcosis in vivo mice model
Source: PLoS One. 2023 Oct 5;18(10):e0292434. doi: 10.1371/journal.pone.0292434 (PMC10553360; doi:10.1371/journal.pone.0292434)
Supplement: Attachment — Submitted filename: Response to Reviewers.doc [file pone.0292434.s004.doc]

**Dear Editor-in-Chief:**

On behalf of my co-authors, I thank you very much for giving us an opportunity to revise our manuscript, we appreciate editors and reviewers very much for their positive and constructive comments and suggestions on our manuscript entitled "**Tumor suppressor p73 induces apoptosis of murine peritoneal cell after exposure to hydatid cyst antigens; a possibly survival mechanism of cystic echinococcosisin vivo mice model**" (PONE-D-23-17969). We have studied reviewers’ comments carefully and tried our best to revise our manuscript according to the comments. All suggestions were separately considered, addressed point-by-point and all of edited changes were highlighted. We hope that the revised form of our manuscript meets the “**PLOS ONE”** journal and would be satisfactory for acceptance.

**Answers to comments of Reviewers**

We would like to thank you very much for the very thoughtful review that helped us greatly to revise the manuscript and to improve its quality.

**Comments to the Author**

1. Is the manuscript technically sound, and do the data support the conclusions?

The manuscript must describe a technically sound piece of scientific research with data that supports the conclusions. Experiments must have been conducted rigorously, with appropriate controls, replication, and sample sizes. The conclusions must be drawn appropriately based on the data presented.

Reviewer #1: Yes

Reviewer #2: Yes

2. Has the statistical analysis been performed appropriately and rigorously?

Reviewer #1: I Don't Know

Reviewer #2: Yes

3. Have the authors made all data underlying the findings in their manuscript fully available?
The [PLOS Data policy](http://www.plosone.org/static/policies.action#sharing) requires authors to make all data underlying the findings described in their manuscript fully available without restriction, with rare exception (please refer to the Data Availability Statement in the manuscript PDF file). The data should be provided as part of the manuscript or its supporting information, or deposited to a public repository. For example, in addition to summary statistics, the data points behind means, medians and variance measures should be available. If there are restrictions on publicly sharing data—e.g. participant privacy or use of data from a third party—those must be specified.

Reviewer #1: Yes

Reviewer #2: Yes

4. Is the manuscript presented in an intelligible fashion and written in standard English?
PLOS ONE does not copyedit accepted manuscripts, so the language in submitted articles must be clear, correct, and unambiguous. Any typographical or grammatical errors should be corrected at revision, so please note any specific errors here.

Reviewer #1: No

Reviewer #2: Yes

**Answer:** Thank you for your attention. The manuscript was checked again for grammar and spelling.

5. Review Comments to the Author

Please use the space provided to explain your answers to the questions above. You may also include additional comments for the author, including concerns about dual publication, research ethics, or publication ethics. (Please upload your review as an attachment if it exceeds 20,000 characters)

**Reviewer #1:**

• The sections "RNA extraction and cDNA synthesis" and "Real-time PCR" could be merged and presented in one section.

**Answer:** We thank the honorable reviewer for their valuable input and suggestion. Sections were merged based on the reviewer's comment.

• In Table 1 please mention the accession number of each gene.

**Answer:** Thank you for your attention. The accession numbers were added.

• In the sentence "After the end of the blocking time, the paper is incubated with the primary antibody mixed and diluted with the blocking solution to a certain amount of β-actin primary antibody (sc-47778, 1:300) for 16 to 18 hours." please mention the specification of all primary antibody which used in this study?

**Answer:** Thank you for your suggestion. This section was revised and it was added.

• In the section "Detection of Caspase-3 activity" please mention the method of total protein extraction. Furthermore, How did the authors evaluate the concentration of extracted proteins?

**Answer:** For detection of Caspase-3 activity, about 10 peritoneal cells were re-suspended with 50 μl of chilled Cell Lysis Buffer (provided in the kit) and incubated on ice for 10 minute. Then the protein extracted from these cells was evaluated according the kit instruction.

• Figure 1 should be edited the y-axis label. I think the relative caspase 3 activity was proper.

**Answer:** Thank you for your attention. It was edited.

Reviewer #2:

 This manuscript entitled “Tumor suppressor p73 induces apoptosis of murine peritoneal cell after exposure to hydatid cyst antigens model'' presents a possibly survival mechanism of cystic echinococcosis in vivo mice model. Importantly, this MS highlights oxidative stress and p73can trigger the apoptosis of murine peritoneal cells through modulator of HF-treated PSCs that is likely one of the hydatid cyst survival mechanisms in vivo mice. As a result, it is a well-organized MS. However, before any proceeding some improvements should be addressed into new version.

Comments
Some parts of the article should be revised in terms of English and grammar.
Abstract:
The term “in vivo” should be “italic” entire paper

**Answer**: It was done and highlighted into new version.

It seems that “Some paraphrasing” should be done. E.g. line 41: “Mice groups were intraperitoneally inoculated with HF and PSC”

**Answer**: It was done and highlighted into new version.

Line 53: “PSC stathis likely” corrected to “PSCs that is likely

**Answer**: It was done and highlighted into new version.

Keywords: the “Hydatidosis” changed to “cystic echinococcosis”

**Answer**: It was done and highlighted into new version.

“Introduction”
Line 60” the “causescystic” corrected to “causes cystic”

**Answer**: It was done and highlighted into new version.

Line 81” After repeating echinococcus once, it should be abbreviated to “ E. granulosus “ in the rest of article.

**Answer**: It was done and highlighted into new version.

-Head and sub-heads of MS should be numbered.

**Answer**: It was done.

- Results and discussion should be written separately.

**Answer**: It was done.

In addition to apoptosis, it is suggested that in the discussion section, the role of innate immunity of TLR 2 and 4 in the survival of the Hydatid cyst in the human body be investigated based recent published articles:

1-<https://link.springer.com/article/10.1007/s00436-018-5850-6>
2- <https://www.sciencedirect.com/science/article/abs/pii/S014795711930133X>

**Answer**: It was done and highlighted into new version.

6. PLOS authors have the option to publish the peer review history of their article ([what does this mean?](https://journals.plos.org/plosone/s/editorial-and-peer-review-process#loc-peer-review-history)). If published, this will include your full peer review and any attached files.
If you choose “no”, your identity will remain anonymous but your review may still be made public.
**Do you want your identity to be public for this peer review?** For information about this choice, including consent withdrawal, please see our [Privacy Policy](https://www.plos.org/privacy-policy).

Reviewer #1: **Yes:** SaeidAfshar

Reviewer #2: No
